# Supplementary material for: Prediction of ACL injury incidence and analysis of key features in basketball players based on multi-algorithm models
Source: PeerJ. 2025 Oct 14;13:e20141. doi: 10.7717/peerj.20141 (PMC12533536; doi:10.7717/peerj.20141)
Supplement: Supplemental Information 1 [file peerj-13-20141-s001.docx]

The MVC testing methodology was based on standard for specific muscles (<https://www.seniam.org/>) as shown on Figure3.11：**A,** Biceps Femoris (long head and short head) MVC: Supine position. Knee joint flexed at 30°, resistance applied at the back of the ankle joint, requiring the subject to flex the knee. **B, C,** Adductor and abductor muscles MVC: Sitting position. Resistance applied at the front of the ankle joint, trunk and thigh at 90°, knee joint at 135°, requiring the subject to extend the knee. **D,** Quadriceps MVC: Supine position. Knee joint at 90° placed at the edge of the bed, resistance applied at the front of the ankle joint, requiring the subject to extend the knee. **E, F,** Gastrocnemius (medial and lateral head) MVC: Supine position. Foot at 90° to the leg, resistance applied at the bottom of the forefoot, requiring the subject to dorsiflex.


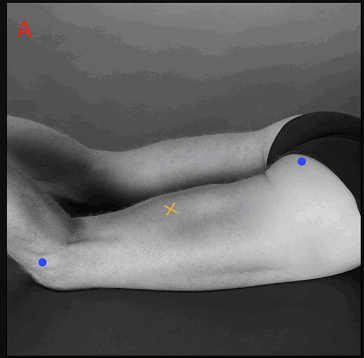

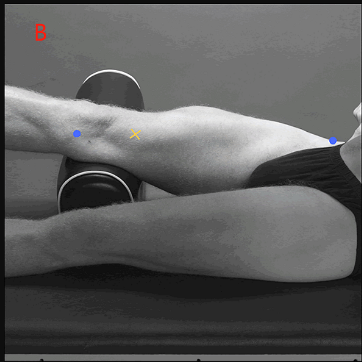

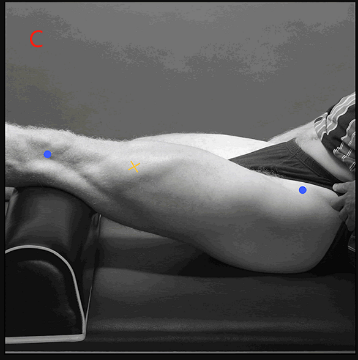


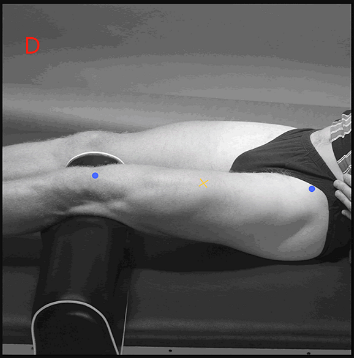

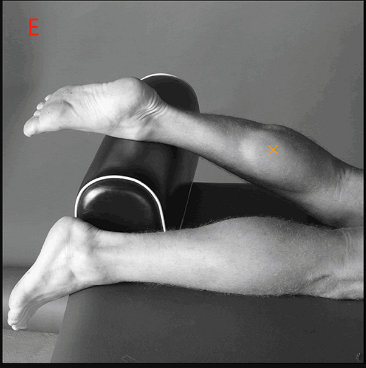

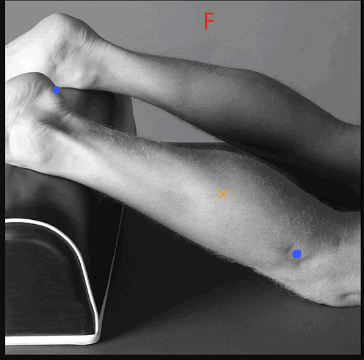


Figure 3.11 MVC testing.
