# Supplementary material for: Prediction of ACL injury incidence and analysis of key features in basketball players based on multi-algorithm models
Source: PeerJ. 2025 Oct 14;13:e20141. doi: 10.7717/peerj.20141 (PMC12533536; doi:10.7717/peerj.20141)
Supplement: Supplemental Information 2 [file peerj-13-20141-s002.docx]

**The optimized parameters for each algorithm.**

Model: SVM, Best Parameters: {'C': 0.1, 'break_ties': False, 'cache_size': 200, 'class_weight': None, 'coef0': 0.0, 'decision_function_shape': 'ovr', 'degree': 3, 'gamma': 0.01, 'kernel': 'rbf', 'max_iter': -1, 'probability': True, 'random_state': None, 'shrinking': True, 'tol': 0.001, 'verbose': False}

Model: Random forest, Best Parameters: {'bootstrap': True, 'ccp_alpha': 0.0, 'class_weight': None, 'criterion': 'gini', 'max_depth': 4, 'max_features': 'auto', 'max_leaf_nodes': None, 'max_samples': None, 'min_impurity_decrease': 0.0, 'min_samples_leaf': 1, 'min_samples_split': 2, 'min_weight_fraction_leaf': 0.0, 'n_estimators': 50, 'n_jobs': None, 'oob_score': False, 'random_state': None, 'verbose': 0, 'warm_start': False}

Model: Logistic regression, Best Parameters: {'C': 10, 'class_weight': None, 'dual': False, 'fit_intercept': True, 'intercept_scaling': 1, 'l1_ratio': None, 'max_iter': 100, 'multi_class': 'auto', 'n_jobs': None, 'penalty': 'l2', 'random_state': None, 'solver': 'lbfgs', 'tol': 0.0001, 'verbose': 0, 'warm_start': False}

Model: XGBoost, Best Parameters: {'objective': 'binary:logistic', 'use_label_encoder': False, 'base_score': 0.5, 'booster': 'gbtree', 'callbacks': None, 'colsample_bylevel': 1, 'colsample_bynode': 1, 'colsample_bytree': 1, 'early_stopping_rounds': None, 'enable_categorical': False, 'eval_metric': None, 'gamma': 0, 'gpu_id': -1, 'grow_policy': 'depthwise', 'importance_type': None, 'interaction_constraints': '', 'learning_rate': 0.1, 'max_bin': 256, 'max_cat_to_onehot': 4, 'max_delta_step': 0, 'max_depth': 3, 'max_leaves': 0, 'min_child_weight': 1, 'missing': nan, 'monotone_constraints': '()', 'n_estimators': 30, 'n_jobs': 0, 'num_parallel_tree': 1, 'predictor': 'auto', 'random_state': 0, 'reg_alpha': 0, 'reg_lambda': 1, 'sampling_method': 'uniform', 'scale_pos_weight': 1, 'subsample': 1, 'tree_method': 'exact', 'validate_parameters': 1, 'verbosity': None}
