# Supplementary material for: Prediction of ACL injury incidence and analysis of key features in basketball players based on multi-algorithm models
Source: PeerJ. 2025 Oct 14;13:e20141. doi: 10.7717/peerj.20141 (PMC12533536; doi:10.7717/peerj.20141)
Supplement: Supplemental Information 8 [file peerj-13-20141-s008.pdf]

ISRCTN18009799 https://doi.org/10.1186/ISRCTN18009799

Prediction of anterior cruciate ligament injury in basketball players using machine learning

**Submission date**<br>22/08/2024	**Recruitment status**<br>No longer recruiting	[ ] Prospectively registered
		[ ] Protocol
**Registration date**<br>22/08/2024	**Overall study status**<br>Completed	[ ] Statistical analysis plan
		[ ] Results
**Last Edited**<br>22/08/2024	**Condition category**<br>Musculoskeletal Diseases	[ ] Individual participant data
		[X] Record updated in last year

Plain English Summary

Background and study aims

This study aims to find out what factors increase the risk of ACL (anterior cruciate ligament) injuries in young adult basketball players. The ACL is a crucial ligament in the knee, and injuries to it can be serious and often require surgery. The researchers also want to see if machine learning, a type of computer technology, can accurately predict who might get injured. By doing this, they hope to identify important risk factors specific to male basketball players, which could help in creating better injury prevention strategies.

Who can participate?

To participate in this study, you must be:

1. Male
2. At least 18 years old
3. Exercising at least 8 hours per week
4. Playing basketball for at least 3 years
5. Not having a knee injury based on a specific test called the Lachman’s test

What does the study involve?

If you join the study, you will undergo several tests to measure factors related to ACL injuries. After the tests, you will be followed for 12 months to see if you sustain an ACL injury during that time. Your data will help in developing and validating a machine learning model to predict ACL injuries in basketball players.

What are the possible benefits and risks of participating?

By participating, you could help advance understanding of ACL injuries in basketball players and contribute to better injury prevention strategies in the future. There may be minimal risks associated with the physical tests, but these will be conducted by trained professionals to ensure your safety.

Where is the study run from?

The study is being conducted at Hospital Universiti Sains Malaysia

When is the study starting and how long is it expected to run for?
October 2021 to April 2024

Who is funding the study?
Investigator initiated and funded

Who is the main contact?
Guo-Long-fei (guolongfei0422@student.usm.my)
Dr Shazlin Binti Shaharudin (shazlin@usm.my).

Contact information

Type(s)
Principal Investigator

Contact name
Prof Shazlin Shaharudin

ORCID ID
http://orcid.org/0000-0001-5583-7631

Contact details
School of Health Sciences,Universiti Sains Malaysia,16150 Kubang Kerian
Kelantan
Malaysia
150000
+60 9-767 7578
shazlin@usm.my

Type(s)
Public, Scientific

Contact name
Dr Longfei Guo

ORCID ID
http://orcid.org/0009-0005-2098-2413

Contact details
Building 7, Wenxingyuan, Qianbeitun Road
Taiyuan
China
30000
+86 15536887299
guolongfei0422@student.usm.my

Additional identifiers

EudraCT/CTIS number
Nil known

IRAS number

ClinicalTrials.gov number
Nil known

Secondary identifying numbers
USM/JEPeM/22040199

Study information

Scientific Title
Prediction of anterior cruciate ligament (ACL) injuries in basketball players using machine learning algorithms: a prospective study

Acronym
B-ACL-ML

Study hypothesis
1. Athlete's profile, Physical function, basketball-specific skills, biomechanics and electromyography are related to ACL injury risk among basketball athletes.
2. The predictive model is valid in distinguishing between male basketball players with and without ACL injuries.

Ethics approval required
Ethics approval required

Ethics approval(s)
Approved 01/09/2022, Jawatankuasa Etika Penyelidikan Manusia Universiti Sains Malaysia (Universiti Sains Malaysia 16150 Kubang Kerian, Kelantan., Kelantan, 15000, Malaysia; +60 9-7673000; jepem@usm.my), ref: USM/JEPeM/22040199

Study design
Single-center observational cohort study

Primary study design
Observational

Secondary study design
Cohort study

Study setting(s)
University/medical school/dental school

Study type(s)
Diagnostic, Prevention, Screening, Efficacy

Participant information sheet
No participant information sheet available

Condition

Prevention of anterior cruciate ligament (ACL) injury in male basketball players

Interventions
In this study, 104 young adult basketball players volunteer to participate. The athletes' profiles, physical functions, basketball-specific skills, biomechanics, and electromyography (EMG) of seven muscles, including the quadriceps, hamstrings, and gastrocnemius, are measured during unanticipated side-cutting maneuvers. After a 12-month follow-up, these data will be compared between those who sustain injuries and those who remain injury-free.

Intervention Type
Behavioural

Primary outcome measure
Measured at baseline and 12 months:
1. The athlete's profile (height, weight, age, level of play, playing position), basketball training record, and self-reported injury history for each participant were recorded
2. Balance testing and joint mobility testing were conducted using YBT and FMS, with a duration of half an hour
3. Biomechanical and synchronized electromyography (EMG) experiments were performed, lasting for two hours
4. Trunk testing was conducted using DLH, strength testing was performed with 1-RM weighted squat and deadlift, explosive strength was assessed using countermovement jump (CMJ), squat jump (SJ), and drop jump (DJ), and agility testing was carried out with the Lane Agility Test lasting for two hours

Secondary outcome measures
There are no secondary outcome measures

Overall study start date
07/10/2021

Overall study end date
03/04/2024

Eligibility

Participant inclusion criteria
1. Male
2. Age over 18 years
3. Exercising ≥8 hours per week,
4. Having played basketball for at least 3 years
5. Having a negative Lachman's knee examination

Participant type(s)
Healthy volunteer

Age group
Adult

Lower age limit

18 Years

Upper age limit
30 Years

Sex
Male

Target number of participants
120

Total final enrolment
114

Participant exclusion criteria
1. Exercise-related or neurological disorders
2. Recent hip or knee surgery or trauma
3. Incomplete data not being analyzed

Recruitment start date
02/09/2022

Recruitment end date
04/04/2023

Locations

Countries of recruitment
China

Study participating centre
Taiyuan University of Technology High Performance Sports Centre
No.18, Xinminyuan Road, Wanbailin District
Taiyuan
China
030024

Study participating centre
Xinzhou Normal University
No.1, Dunqi East Street
Xinzhou
China
034000

Sponsor information

Organisation
Hospital Universiti Sains Malaysia

Sponsor details
Health Campus, 16150 Kubang Kerian, Kelantan
Kota Bharu
Malaysia
15000
+60 09-767 3000
hospitalusm@usm.my

Sponsor type
University/education

Website
https://hospital.usm.my/

ROR
https://ror.org/0090j2029

Funder(s)

Funder type
Other

Funder Name
Investigator initiated and funded

Results and Publications

Publication and dissemination plan
Planned publication in a peer-reviewed journal

Intention to publish date
04/12/2025

Individual participant data (IPD) sharing plan
Individual participant data will not be provided due to privacy concerns

IPD sharing plan summary
Data sharing statement to be made available at a later date
